# Wound healing effects of 80% methanol and solvent fractions of the leaf of *Cordia Africana Lam:* (Boraginaceae) against *wound models in* mice

**DOI:** 10.1371/journal.pone.0331377

**Published:** 2025-09-12

**Authors:** Jemal Abdela, Fuad Adem, Ahmedmenewer Abdu, Mohammed Yusuf, Abenezer Aklog Wondimu, Mohammed Abdurke Kure, Mekonnen Sisay, Burka Mohammedsani, Monas Kitessa, Abraham Nigussie Mekuria

**Affiliations:** 1 Department of Pharmacology and Toxicology, School of Pharmacy, College of Health and Medical Sciences, Haramaya University, Harar, Ethiopia; 2 Department of Pharmacy, College of Health and Medical Sciences, Madda Walabu University, Goba, Ethiopia; 3 School of Medical Laboratory Sciences, College of Health and Medical Sciences Haramaya University, Harar, Ethiopia; 4 Department of Clinical Pharmacy and Pharmacy Practice, School of Pharmacy, College of Health and Medical Sciences, Haramaya University, Harar, Ethiopia; 5 School of Midwifery, College of Health and Medical Sciences, Haramaya University, Harar, Ethiopia; 6 Department of Pharmacology and Toxicology, School of Pharmacy, College of Medicine and Health Sciences, Wollo University, Dessie, Ethiopia; 7 Department of Surgery, School of Medicine, College of Health and Medical Sciences, Haramaya University, Harar, Ethiopia; Bowen University, NIGERIA

## Abstract

**Background:**

Chronic wounds present a major health challenge, and traditional remedies are frequently employed in developing nations for wound treatment. This study sought to assess the safety and effectiveness of various solvent extracts from *Cordia africana* using excision and incision wound models in mice.

**Methods:**

The dried, coarsely ground 500 g of plant leaves were separately macerated three times with 80% methanol, absolute methanol, chloroform, and aqueous water. Acute dermal toxicity was assessed by applying 2000 mg/kg of the 10% w/w of the solvent extracts. In both wound models, animals were randomly divided into four groups of five mice each. Group I received a simple ointment and served as the negative control. Group IV was treated with nitrofurazone (0.2%) ointment as the positive control. Groups II and III were treated with 5% w/w and 10% w/w ointments of the solvent extracts, respectively.

**Results:**

The acute dermal toxicity studies demonstrated that the plant leaf extracts are safe, as there were no signs of toxicity observed. In the excision wound model, the application of 5% and 10% ointments of the 80% methanol extract resulted in significant (*p* < 0.05) wound contraction compared to the negative control group, starting from day 13 and day 5, respectively. Similarly, 10% ointments of both absolute methanol and chloroform extracts significantly (**p* *< 0.05) reduced wound size starting from day 7. Furthermore, the time required for complete epithelization was significantly shorter (**p* *< 0.05) for the 80% methanol, chloroform, and absolute methanol extracts compared to the negative control. The chloroform fraction and 10% ointment of the 80% methanol extracts also significantly increased hydroxylproline content. The 5% and 10% chloroform extract ointments (66.02% and 72.20%, respectively) and the 10% ointment with an 80% methanol extract (55.49%) significantly improved the tensile strength of the mice skin (**p* *< 0.05) compared to the group treated with a simple ointment (1.6%).

**Conclusion:**

The study revealed that *Cordia africana* leaf extracts possess significant wound-healing properties, supporting its traditional medicinal use. It was found that using less polar solvents is more effective in extracting components with enhanced wound-healing effects.

## Introduction

The global burden of wounds, which refers to disruptions or damage to the normal structure and function of living tissue, is increasing due to a rise in injuries, an aging population, and the prevalence of diabetes, respiratory diseases, and poor nutrition. [1,2]. Historically, traditional herbal medicines have been widely used for wound healing, earning cultural acceptance and trust from people worldwide [3].

Numerous ethnomedicinal studies in Africa have documented plants traditionally used for wound healing. Yet wound healing properties and mechanisms of action of a few plants have been explored, and much are remains to be understood in the pursuit of discovering newer, more effective, and safer treatments [4,5]. *Cordia africana* Lam (C. Africana) is a versatile tree species from the Boraginaceae family, widely distributed across tropical Africa. It is known by various local names in Ethiopia: “wanza” in Amharic, “waddessa” in Afan Oromo, “awhi” in Tigrigna, and widely referred to as East African Cordia, large-leafed Cordia, or Sudan teak in English [6]. This tree is distinguished by its large leaves and is highly valued for its diverse applications, including traditional medicine, timber production, and ecological importance [6].

The ethnomedicinal use of *C. africana* in treating various diseases is backed by numerous experimental studies. Extracts from different parts of the plant have shown antimicrobial [7,8], anti-inflammatory [9], antioxidant [10], antimalarial [11], anthelmints [12], antidiarrheal [13], and antinociceptive properties [14]. Furthermore, ethnobotanical studies have highlighted the traditional use of the plant’s leaves for treating burns and wounds. For example, in Ethiopia, crushed leaves, either alone or mixed with butter, are applied to wound and burn sites [15,16]. The traditional use of *C. africana* for wound healing has not yet been scientifically validated in live models. Therefore, this study aimed to assess the wound healing properties of *C. africana in vivo* using excision and incision models in mice.

## Methods

### Plant material

The fresh leaves of *C. africana* was collected from Harar town located 500 km east of Addis Ababa, Ethiopia. The plant was identified by a botanist and a voucher specimen (JA03/21) was deposited at the Herbarium, College of Natural and Computational Sciences, Haramaya University for future reference.

### Experimental animals

Healthy adult Swiss albino mice of both sexes, weighing 25–30 g and aged 6–8 weeks, were obtained from the animal facility at the School of Pharmacy, College of Health and Medical Sciences, Haramaya University. The animals were housed in standard cages under controlled conditions of 25 ± 2°C, 55 ± 5% relative humidity, and a 12-hour light/dark cycle. The animals were given unrestricted access to a standard pellet diet and water throughout the study. All experiments were performed in full adherence to internationally recognized guidelines for the care, use, and handling of laboratory animals [17].

### Extraction of plant material

The leaves of *C. africana* were thoroughly washed under running water, cut into smaller pieces, and left to air-dry in the shade for a period of three weeks. Five hundred grams of coarsely ground dried leaves were separately macerated in 80% methanol, absolute methanol, chloroform, and distilled water for three days in conical flasks with regular shaking and stirring. The extracts from each solvent were filtered (Whatman No. 1) and the remaining marcs were re-macerated twice to obtain maximum yield. The extracts were concentrated at 40°C under reduced pressure using a rotary evaporator, followed by complete drying through freeze-drying with a lyophilizer. The dried extracts were weighed to determine the yield and then stored in a refrigerator for later use.

### Preliminary phytochemical screening

The standard procedures were employed to qualitatively analyze the various solvent extracts of C. africana leaves for their phytochemical constituents [18].

### Ointment formulation

The formula in Table 1, described in the British Pharmacopoeia [19], was used to prepare a simple ointment base for plant extract formulations.

**Table 1:** Formula used for preparation of the ointment.

Ingredients	Master formula	Reduced formula
Wool fat	50 g	10 g
Hard paraffin	50 g	10 g
White soft paraffin	850 g	170 g
Cetostearyl alcohol	50 g	10 g
	1000 g	200 g

Each 200 g ointment preparation was formulated using a simplified version of the master formula (Table 1). The formulations included 5% w/w and 10% w/w of the extracts, along with a simple ointment without the extract, which served as the control. The ointment base ingredients were combined, gently heated with continuous stirring until a uniform mixture was achieved, and then stirred further until fully cooled. To prepare a medicated ointment using different extracts, 10 g and 20 g of the extracts were thoroughly mixed with 190 g and 180 g of the ointment base, respectively. The mixing was performed through levigation on an ointment slab to achieve a uniform consistency and a smooth texture [20]. To prepare the control ointment, 200 g of the base were measured and processed similarly, but without the addition of an active ingredient.

### Acute dermal toxicity

The study involved five randomly selected female mice for each extract. Before testing, the mice were acclimated to laboratory conditions for five days. Twenty-four hours before applying the ointment containing the extracts, the hair on the back trunks of the mice was shaved. The ointment, prepared at a 10% (w/w) concentration from each extract, was applied to the shaved areas, which covered approximately 10% of the animals’ total body surface. The ointment was administered at a controlled dose of 2000 mg/kg [21].

### Grouping and dosing of animals

The experiment utilized both excision and incision models, with mice randomly divided into four groups of five animals each. Group I, the negative control, was treated with a standard ointment. Groups II and III were administered ointments containing solvent extracts at concentrations of 5% and 10%, respectively. Group IV, serving as the positive control, was treated with a 0.2% nitrofurazone ointment.

### Excision wound model

**Fig 1:**
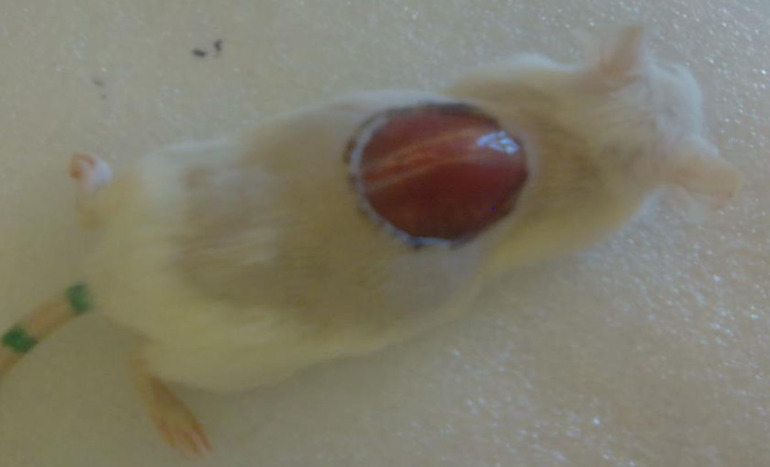
Excision wound on day 0.

The mice were anesthetized subcutaneously with ketamine (80 mg/kg) and diazepam (5 mg/kg) before wound creation [22]. The animal’s back hair was shaved using an electric trimmer, and a 300 mm² section of skin, marked with a fine permanent marker and surgically excised with sterilized scissors and forceps [23]. The day on which the wounds were inflicted was designated as day 0 (Fig 1). The ointments were gently applied once daily, starting 24 hours after the wound was created, according to the specified groupings, and continued until the injury was fully healed. The wound contraction progress, epithelization duration, and hydroxyproline levels were assessed. The extent of wound contraction was evaluated in a percentage every two days until the wound fully closed [24,25].

### Measurement of wound contraction

The progression of wound healing was evaluated by outlining wound areas on a transparency sheet using a permanent marker. The measured surface area was then utilized to calculate the percentage of wound contraction, with the initial wound size designated as 100%, as described below [26].


$$\%\text{Wound contraction}=\frac{(\text{Initial wound size}-\text{Specific day wound size})\text{X 100}}{\text{Initial wound size}}$$

#### Gross epithelization time measurement.

The time is assessed by tracking the number of days required for the scabs to naturally fall off, leaving no exposed wound tissue behind [27].

#### Hydroxyproline content determination.

**Fig 2:**
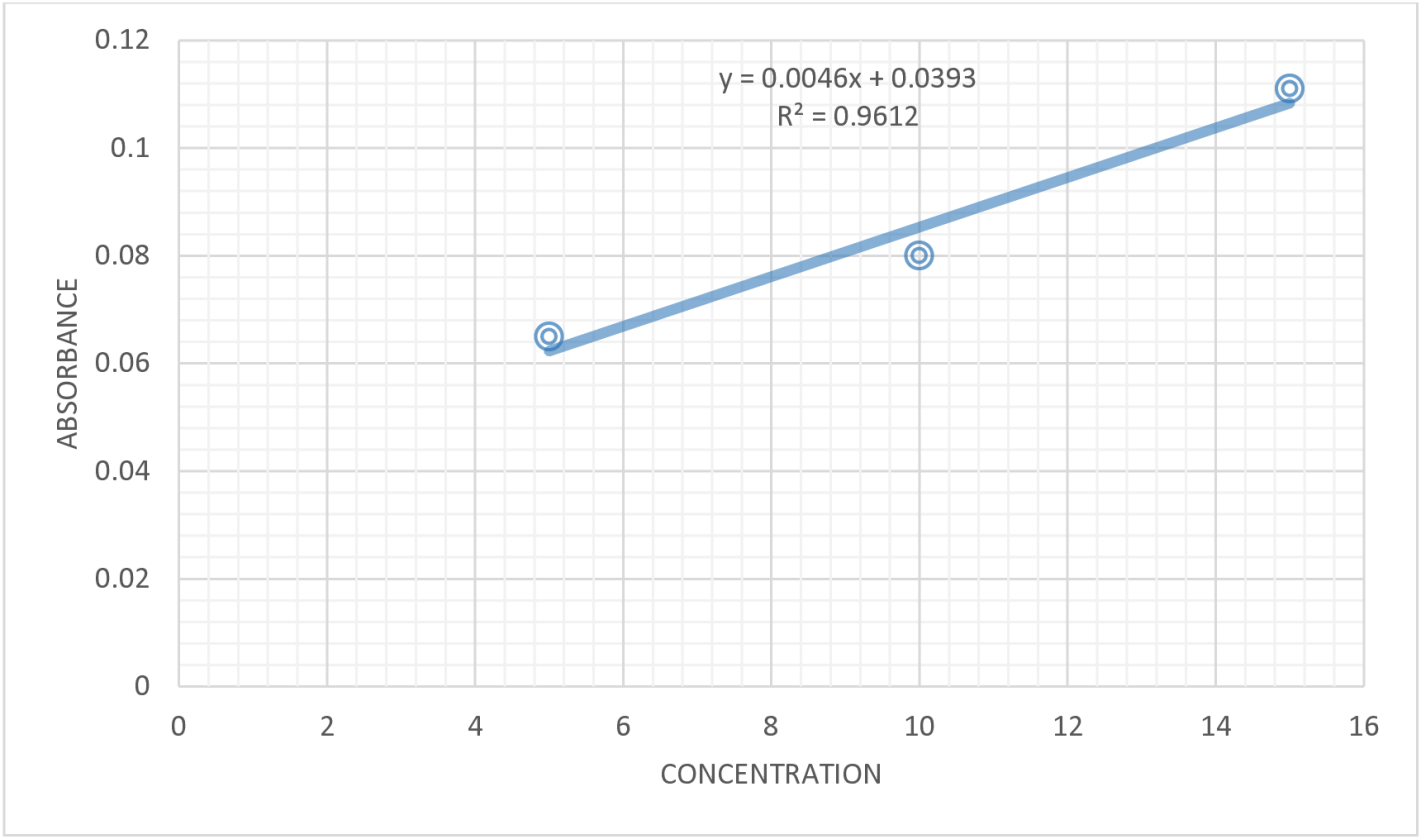
Calibration curve for standard hydroxyproline.

To measure the hydroxyproline content in mouse tissue, a circular wound with an approximate area of 300 mm² was created using the excision wound model procedure. The wounds underwent a 10-day treatment through the topical application of ointments, following the method described by Kokane DD, *et al* [23]. On the 11^th^ day, the animals were euthanized and wound tissue was surgically excised, weighed, and preserved in 10% formalin in a refrigerator. On the experiment day, the tissue was dried in an oven at 60°C for 12 hours, and the new dry weight was recorded. The dried tissue was hydrolyzed in sealed glass tubes with 6 N HCl at 110°C for 24 hours. The resulting hydrolysate was neutralized to pH 7, following the method outlined by Sanwal R and Chaudhary AK [28]. From each tissue hydrolysate, 1 ml of the supernatant was extracted. The absorbance of the hydrolysate was measured using the same procedure utilized for the standard hydroxyproline solution. The hydroxyproline content in the samples was calculated using the equation derived from the calibration curve (Fig 2).

### Incision wound model

**Fig 3:**
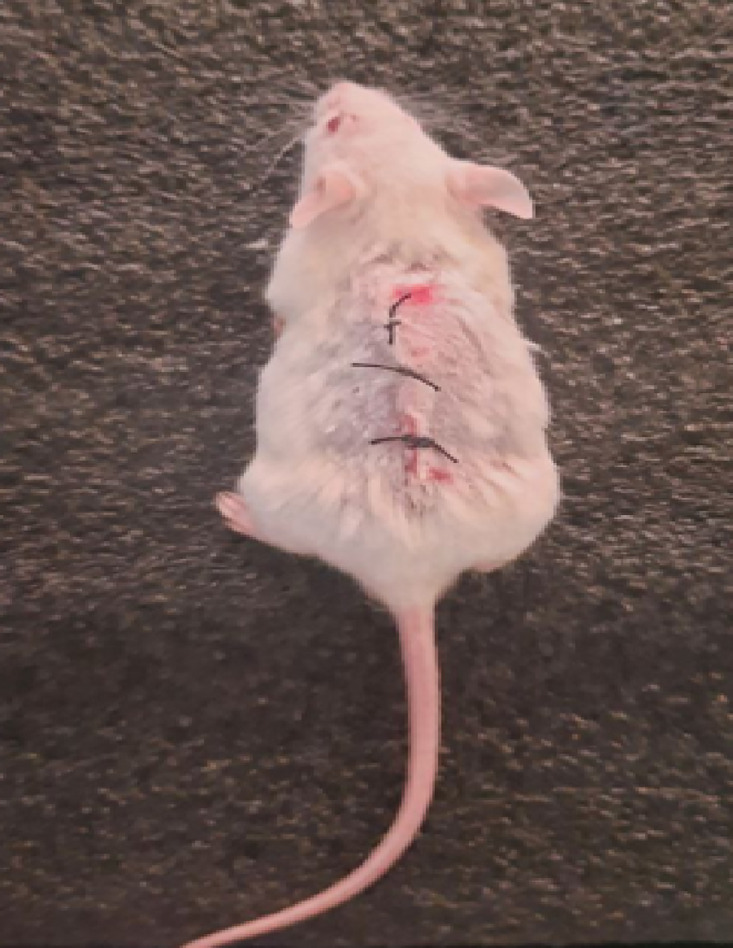
Incision wound on day 0.

The anesthesia procedure was conducted using the same method outlined in the excision wound model, and the dorsal fur of the mice was shaved with a shaving machine. A 3 cm longitudinal paravertebral incision was made through the skin and underlying cutaneous tissue. The separated skin was then sutured at 1 cm intervals using surgical thread (No. 000) and a curved needle (no. 11) (Fig 3). The sutures on both sides of the wound were firmly tightened to guarantee proper wound closure [29]. The experimental animals were treated in a manner similar to the excision wound experiments, except for the fifth group, which did not receive any material application. Sutures were removed on the eighth day post-incision, and the treatment was continued until the ninth day [30].

#### Measurement of tensile strength.

**Fig 4:**
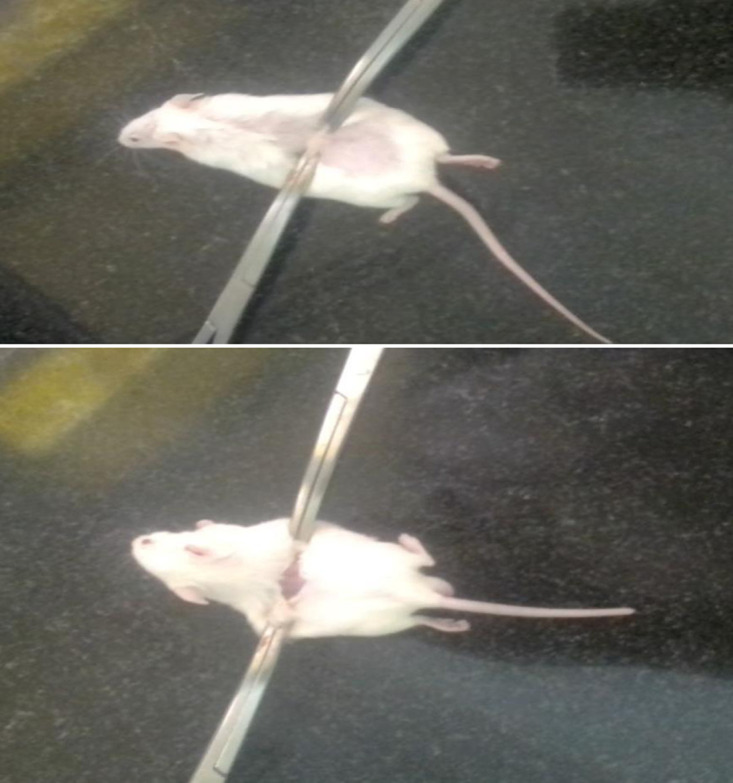
Water flow technique for the measurement of tensile strength of skin.


As illustrated in Fig 4, tensile strength measurements were performed on the 10th day post-wounding using the continuous water flow technique [31]. The tensile strength percentage was calculated for both the extract and the reference drug, compared to the negative control treated with a simple ointment. Furthermore, the tensile strength percentage of the negative control treated with a simple ointment was also calculated relative to the untreated negative control. These calculations followed the formula outlined by Thakur R, Jain N, Pathak R and Sandhu SS [32].

$$\text{Tensile strength}(\text{TS})ofextract\%=\frac{TSextract-TSs.o}{TSs.o}x\text{100}$$

$$\text{Tensile strength}(\text{TS})ofreference\%=\frac{TSreference-TSs.o}{TSs.o}x\text{100}$$

$$\text{Tensile strength}(\text{TS})ofS.O\%=\frac{TSs.o-TSl.u}{TSl.u}x\text{100}$$

Where s.o is simple ointment, and l.u is the tensile strength of left untreated.

### Methods of scarification and alleviating the suffering of animals

Before excision and incision procedures, as highlighted under the excision procedure, animals were anesthetized with ketamine and diazepam to reduce the sense of pain associated with the procedure. After the completion of the experimental procedures , the animals were collected and put into a desiccator and anesthetized with a high dose of isoflurane at a concentration of 5%. The animals became immobile, and death was ensured by cervical dislocation of each mouse and discarded in the disposal area prepared for this purpose.

### Statistical analysis

Data are expressed as mean ± standard error of the mean (SEM) for each group. Statistical analyses were conducted using one-way analysis of variance (ANOVA), followed by Tukey’s post hoc test for multiple group comparisons. Data normality was assessed through histograms and Q-Q plots. A *p*-value of less than 0.05 was considered statistically significant. All analyses were performed using SPSS software, version 20.0.

### Ethical consideration

Permission to conduct a study was obtained from the Animal Research Ethics and Review Committee of the College of Health and Medical Sciences with a Ref. No. ARERC/226/2021. The rodents were given one week to adapt to the laboratory environment before the start of the experiment. The animals were handled and cared for by following the internationally recognized guidelines [33].

## Results

### Percentage yield of extraction

The extraction with 80% methanol yielded the highest percentage (26%) from the leaves of *C. africana*, as shown in Table 2.

**Table 2:** Yield and physical properties of the different solvent extracts of the leaves of Cordia africana.

Types of extract	Nature of extract	Color of extract	Actual yield (g)	% Yield (w/w)
80% ME	Slightly gummy	Light black	130^*^	26%
ME	Slightly gummy	Light black	80^*^	16%
CE	Very gummy	Dark black	10.23^*^	2.04%
AE	Slightly gummy	Light brown	55^*^	11%

Note: ^*^ Yield obtained from 500 g of coarsely powdered *Cordia africana* leaf. 80ME, 80% methanol extract; AE, Aqueous extract; CE, Chloroform extract; ME, Methanol extract

### Acute toxicity

**Fig 5:**
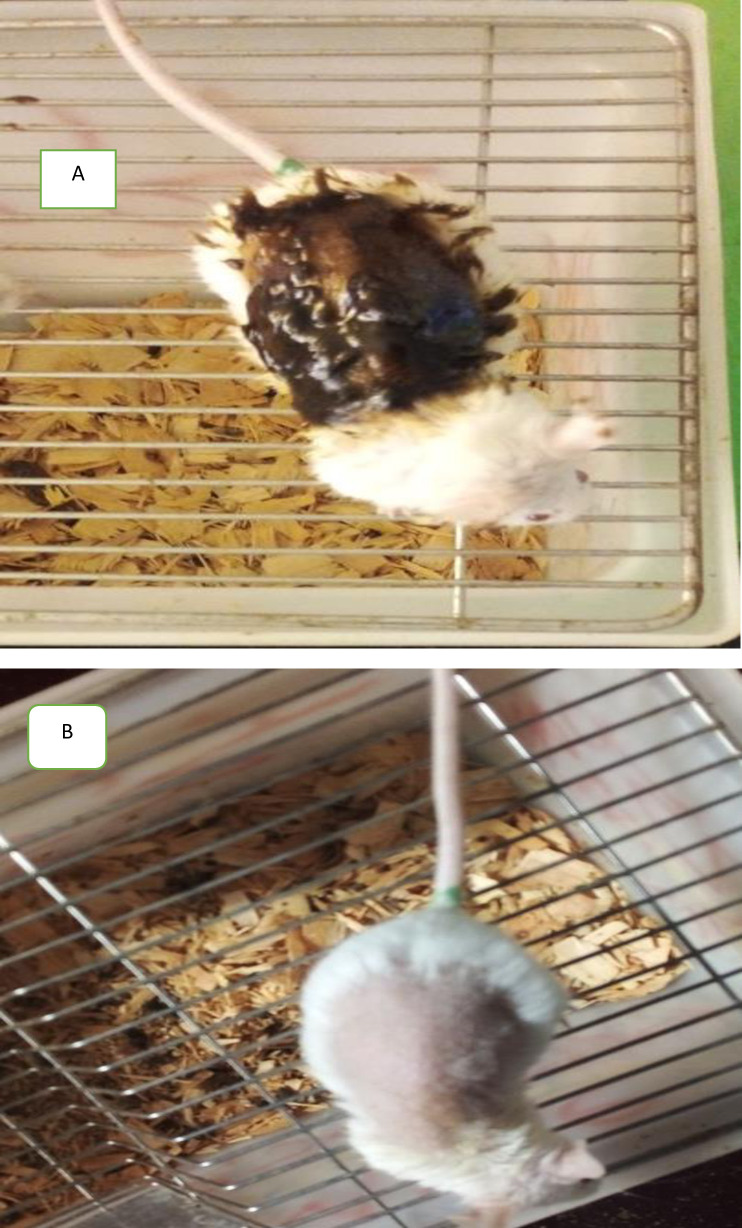
Photographs of the mouse skin during acute skin toxicity. A = indicates when the plant extract is applied to the shaved dorsal part, B = indicates absence of any reaction on the skin after the plant extracts applied.

The maximum concentration of each solvent extract ointment (10% w/w), applied at a limit dose of 2000 mg/kg of body weight, and was found to be safe. After 24 hours of application, the treated site showed no signs of inflammation (Fig 5). Additionally, no noticeable signs or symptoms were observed during the 48-hour monitoring period. Furthermore, no evidence of toxicity or mortality was recorded throughout the 14-day cage-side observation period.

### Excision wound model

#### Wound contraction.

**Fig 6:**
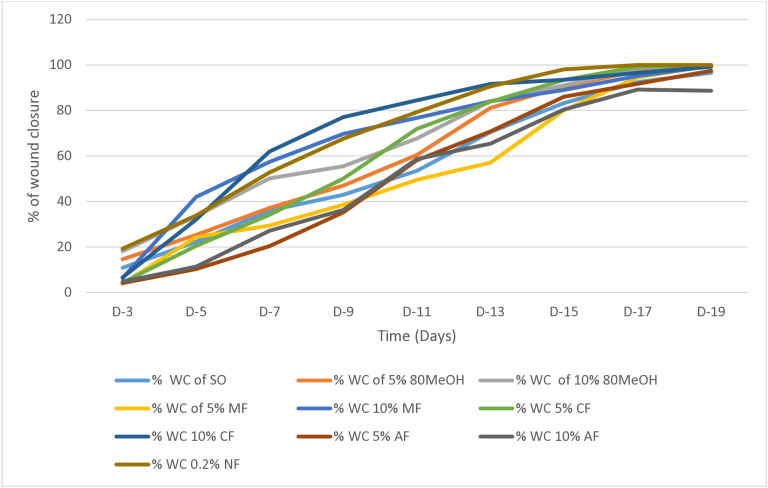
Effects of 5% and 10% ointment of 80% ME, ME, CE and AE extracts of Cordia Africana leaf on the percentage wound closure of excision wound model.

Table 3 and Fig 6 illustrate the wound contraction effects of 5% and 10% *C. africana* extract ointments, simple ointment base, and 0.2% nitrofurazone. Compared to the negative control, the 10% hydro-methanolic (80% methanol) and chloroform extract ointments demonstrated significant wound contraction (*p* < 0.001) starting from days 5 and 7, respectively. In contrast, the lower concentrations of chloroform and hydro-methanolic ointments showed significant contraction beginning on the 11^th^ day (*p* < 0.001) and 13^th^ day (*p* < 0.05), respectively, when compared to the negative control. However, throughout the treatment period, both concentrations of the aqueous fraction did not produce statistically significant effects compared to the control group (Table 3).

**Table 3:** Effect of topical application of the different solvent extracts of the Cordia africana leaves on wound contraction of excision wound model in mice.

Wound area (mm2) post-wounding days										
	SO	5% 80 ME	10% 80 ME	5% ME	10% ME	5% CE	10% CE	5% AE	10% AE	0.2% NF
**Day 3**	267.44 ± 3.95	256.14 ± 3.03	245.69 ± 8.47	288.53 ± 5.77	280.90 ± 6.21	287.04 ± 6.54	280.07 ± 7.05	287.61 ± 6.31	285.73 ± 9.99	242.34 ± 5.57^a*d*e*f**g*h*i*^
**Day 5**	233.28 ± 2.92	223.98 ± 2.20	199.44 ± 3.11^a**b*^	226.91 ± 28.29	174.10 ± 13.42	238.68 ± 11.41	203.77 ± 20.68	269.16 ± 6.21	266.06 ± 13.80	198.59 ± 7.24^a2b*h**i*^
**Day 7**	191.85 ± 2.75	188.54 ± 3.46	149.69 ± 5.02^a**b**^	211.80 ± 25.42	127.73 ± 11.87^a*d*^	197.61 ± 9.81	114.02 ± 15.70 ^a**f**^	238.95 ± 5.67	218.49 ± 20.54	141.59 ± 4.99^a**b**f*h**i*^
**Day 9**	171.26 ± 1.34	158.93 ± 5.50	133.63 ± 8.05^a**b*^	184.39 ± 22.19	90.87 ± 6.79 ^a*d**^	149.72 ± 13.02	68.71 ± 7.14 ^a**f**^	194.36 ± 10.00	191.25 ± 19.30	97.16 ± 7.17^a**b**c*d**f*h**i*^
**Day 11**	139.44 ± 3.79	118.84 ± 5.12	96.90 ± 8.98^a*^	151.38 ± 27.07	69.91 ± 10.59 ^a*d*^	84.28 ± 22.55^a**^	46.69 ± 3.42^a**^	125.69 ± 18.18	124.15 ± 23.20	62.23 ± 10.87^a**b**c*d*^
**Day 13**	88.36 ± 2.37	56.70 ± 2.11 ^a*^	47.81 ± 6.47 ^a**^	128.83 ± 19.43	48.03 ± 6.52^d*^	48.36 ± 8.11^a**^	24.89 ± 5.88^a**^	87.74 ± 14.04	103.87 ± 25.21	28.15 ± 5.61^a**b*c*d*i*^
**Day 15**	50.33 ± 3.61	28.20 ± 1.75^a*^	26.96 ± 4.60 ^a**b*^	59.84 ± 13.58	32.85 ± 6.47	19.79 ± 2.77^a**^	19.53 ± 4.64 ^a**^	41.80 ± 13.75	58.76 ± 12.59	5.84 ± 1.06 ^a**b*c*d*f*g*i*^
**Day 17**	22.73 ± 0.67	7.95 ± 1.27 ^a**^	4.27 ± 1.29 ^a**^	16.33 ± 6.75	14.59 ± 3.89	2.37 ± 1.48^a**^	10.22 ± 2.84 ^a**f*^	24.74 ± 9.42	7.65 ± 3.96	0.00 ± 0.00 ^a**b**c*d*f*i*^
**Day 19**	10.37 ± 0.65	2.15 ± 0.95 ^a**^	0.00 ± 0.00 ^a**^	0.43 ± 0.43^a**^	1.26 ± 1.26^a**^	0.00 ± 0.00^a**^	2.30 ± 2.30^a*^	17.60 ± 0.51	17.60 ± 0.40	0.00 ± 0.00 ^a**b*h*^

Values are mean ± SEM (n = 5); analysis was done using one-way ANOVA followed by Tukey HSD post hoc test, ^a^ compared with negative control (SO), ^b^compared with 5% 80 ME, ^c^compared with 10% 80 ME, ^d^compared with 5% ME, ^e^compared with 10% ME, ^f^compared with 5% CE, ^g^compared with 10% CE, ^h^compared with 5% AE, ^i^compared with 10% AE,^*^p < 0.05, ^**^p < 0.001. 80 ME, 80% Methanol extract; ME, Methanol extract; AE, Aqueous extract, CE, Chloroform extract; AE, Aqueous extractAE, Aqueous extractddfghhAAAAE

#### Epitheliazation period.

**Fig 7:**
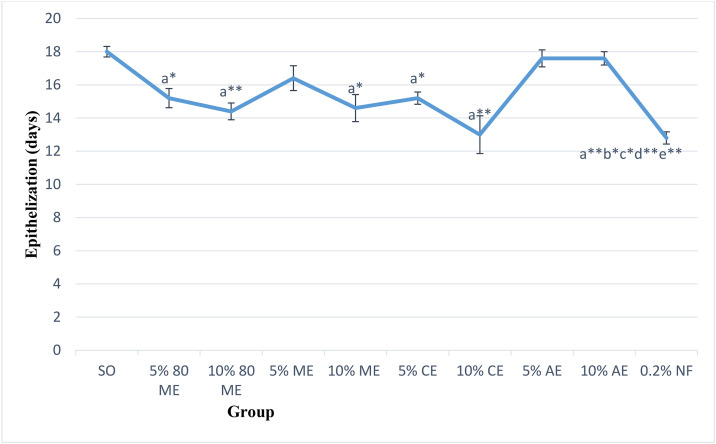
Effects of 5% and 10% ointments of the extracts of *Cordia africana* leaf on the epithelization period. Values are mean ± SEM (n = 5); analysis was done using one-way ANOVA followed by Tukey HSD post hoc test, ^a^ compared with negative control/SO, ^b^compared with 5% 80ME, ^c^compared with 5% ME, ^d^compared with 5% AE, ^e^compared with 10% AE, ^*^p < 0.05, ^**^p < 0.001. ME, methanol extract; CE, chloroform extract; AE, aqueous extract; SEM, standard mean of error; NF, nitrofurazone.

Fig 7 presents the epithelization period for excision wounds treated with various extracts and control formulations. Mice treated with both concentrations of the hydro-methanolic and chloroform extracts, along with the 10% absolute methanol extract and the positive control (0.2% nitrofurazone), demonstrated significantly shorter epithelization periods compared to the negative controls. The shortest epithelization time (28.9%) was observed with nitrofurazone (**p* *< 0.001), followed by the 10% chloroform extract (27.8%) and the 10% hydro-methanolic extract (20%) (*p* < 0.001).

### Hydroxyproline content

**Fig 8:**
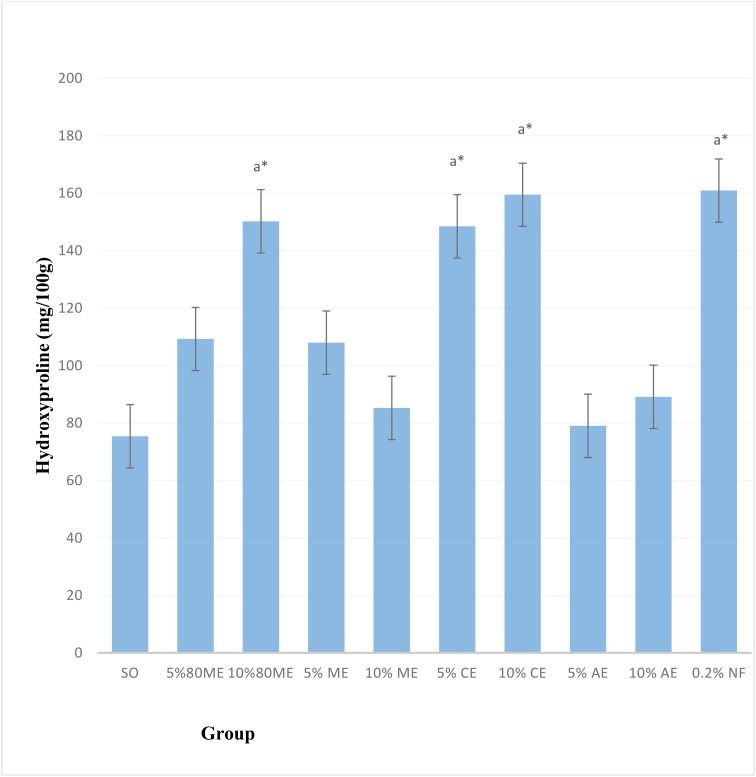
Effects of 5% and 10% ointments of the extracts of *Cordia africana* leaf on the hydroxyproline content. Values are mean ± SEM (n = 5); analysis was done using one-way ANOVA followed by Tukey HSD post hoc test, ^a^ compared with negative control (simple ointment), ^*^p < 0.05. ME, methanol extract; CE, chloroform extract; AE, aqueous extract; SEM, standard mean of error; NF, nitrofurazone.

As illustrated in Fig 8, the hydroxyproline content significantly increased in mice treated with 5% and 10% chloroform extracts, 10% hydro-methanolic extract, and 0.2% nitrofurazone compared to the negative controls. Among these, 0.2% nitrofurazone showed the highest percentage of increase (113.3%), followed closely by the 10% chloroform extract (111.4%) and the 10% hydro-methanolic extract (99.1%) (**p* *< 0.05). However, no noticeable differences were observed between the extracts and the positive control.

### Incision wound model

**Fig 9:**
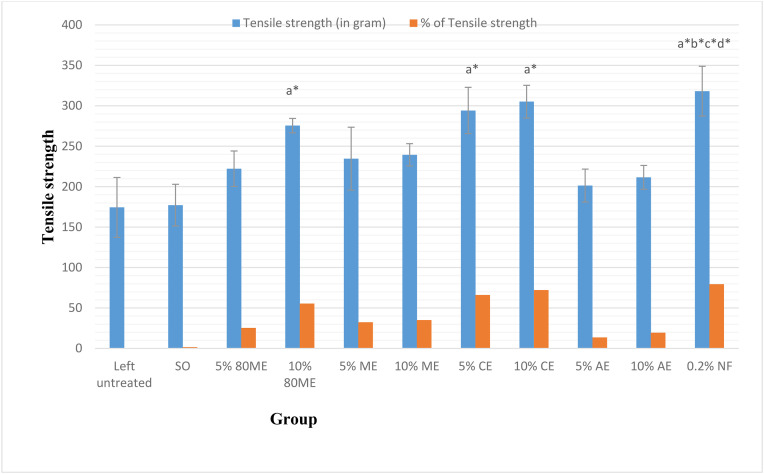
Effect of topical application of 5% and 10% ointments of extracts of *C. africana* leaves on tensile strength of incision wound model. Values are mean ± SEM (n = 5); analysis was done using one-way ANOVA followed by Tukey HSD post hoc test, ^a^compared with negative control, ^b^compared with 5% 80 ME, ^c^compared with 5% AE, ^d^compared with 10% AE, ^*^p < 0.05. 80 ME, 80% methanol extract; ME, methanol extract; CE, chloroform extract; AE, aqueous extract; NF-nitrofurazone.

As shown in Fig 9, a significant increase in mean tensile strength was observed in animals treated with 10% (72.20%) and 5% (66.02%) chloroform extract ointments, as well as 10% hydro-methanolic extract ointment (55.49%), compared to the negative control group (*p* < 0.05). These results were comparable to the positive control group (79.5%).

### Phytochemical screening

Phytochemical analysis of various solvent extracts from *C. africana* leaves indicated the presence of bioactive compounds, as detailed in Table 4.

**Table 4:** Phytochemical screening of the different solvent extracts of the leaves of Cordia africana.

Phytochemical screening	CE	80ME	ME	AE
Flavonoids	–	+	+	+
Saponins	–	+	+	+
Phenols	–	+	–	+
Tannins	+	+	+	+
Terpenoids	+	–	+	+
Steroids	+	–	+	–
Phytosterols	–	–	+	+
Cardiac glycosides	+	+	–	+
Alkaloids	–	+	–	+
Anthocyanin	–	–	–	+
Quinones	–	+	–	+
Reducing sugar	+	–	+	–

+, the presence of phytochemical constituents; -, the absence of phytochemical constituents 80 ME, 80% Methanol extract; AE, Aqueous extract; CE, Chloroform extract; ME, Methanol extract

## Discussion

For centuries, medicinal plants have been utilized to treat a wide range of skin ailments and dermatological disorders, particularly cuts, wounds, and burns. This practice has been prevalent since ancient times and has stood the test of time as an effective approach to addressing such conditions [3,34]. The safety and effectiveness of various medicinal plants and their derivatives as wound-healing agents have been subject to scrutiny, despite their widespread usage [35]. In Ethiopia and Tanzania, the leaves of *C. africana* Lam are crushed into a powder and combined with butter to create a remedy for treating burns and wounds [16,36]. To closely mimic the method of traditional application and extraction [37], different solvents were used, from polar to nonpolar, to confirm the traditional claims of plant leaf extracts in wound healing. The study assessed the safety and effectiveness of 80% methanol, absolute methanol, chloroform, and aqueous extracts from *C. africana* leaves using excision and incision wound models.

The dermal toxicity evaluation revealed that the plant extract is safe at the maximum concentration (10%) of all solvent extract ointments prepared and applied at a dose of 2000 mg/kg. This finding aligns with a previous report on the safe use of crushed plant leaves in the treatment of wounds [15].

In excision wound model, higher concentrations (10%) of the hydro-methanolic and chloroform extract ointments of the plant showed a significant wound contraction starting from the first weeks of treatment. The result substantiates the beneficial effects of the *C. africana* leaf in wound healing as claimed by traditional healers when applied by crushing and mixing it with butter [16]. The findings suggested that pharmacologically active components required for wound healings are better extracted with nonpolar solvent as evidenced from chloroform ointment effects and more likely to be concentrated in 10% ointment formulation which revealed rapid wound closure compared to 5% ointment formulation. Increased wound contraction has been shown to reduce wound size and minimize the amount of extracellular matrix needed for tissue repair during the proliferative phase of wound healing. It also promotes re-epithelialization by reducing the distance migrating keratinocytes must traverse [2,38]. The notable enhancement in wound contraction observed with the chloroform and hydro-methanolic extracts of *C. africana* leaves highlights their potential pro-wound healing properties, as contraction is a crucial factor in the healing process of wounds healing by secondary intention [39]. The precise mechanism through which the extracts promote wound contraction is not yet fully understood. However, it is plausible that this effect is associated with the plant’s antioxidant, anti-inflammatory, and antimicrobial properties [8,10].

The study revealed that animals treated with ointments containing 10% chloroform exhibited significantly shorter epithelization times, followed by those treated with hydro-methanolic extracts. Epithelization, a critical phase in wound healing, involves the proliferation and migration of epithelial cells at the wound edges, a process stimulated by growth factors [40]. The reduced epithelization period observed with the extracts may be due to enhanced epithelial cell proliferation or improved cell viability [28,41] in the injured site. These findings suggest that the observed effects, such as enhanced epithelization and increased wound contraction upon the application of the ointment containing plant extracts, could be linked to the plant’s ability to enhance collagen production, potentially through its impact on hydroxyproline content. Enhanced collagen synthesis would ultimately contribute to the closure of the wound [42]. The lack of significant effects observed in various models despite the presence of multiple phytochemical components in aqueous fraction may be ascribed to its lower concentration of active constituents responsible for wound healing effects.

The analysis of hydroxyproline content demonstrated significantly higher levels in animals treated with 10% hydro-methanolic and chloroform extract ointments, showing no notable difference compared to the standard drug (0.2% nitrofurazone). This indicated that the extracts effectively promoted wound healing by enhancing collagen synthesis and deposition at the wound site. The elevated hydroxyproline levels reflect increased cellular proliferation, leading to improved collagen production [43].

The 10% chloroform and hydro-methanolic extract ointments showed a significant increase in mean tensile strength compared to the negative control groups. This enhancement in tensile strength indicates that the plant extracts contribute to improved tension resistance and overall quality of the repaired tissue [44]. Various factors may contribute to this, such as fibroblast proliferation, migration to the wound site, collagen synthesis and maturation, angiogenesis, and fiber stabilization. Together, these effects improve circulation, ensuring the efficient delivery of oxygen and nutrients essential for wound healing. Furthermore, this process strengthens and enhances the integrity of the wound matrix [45,46].

The phytochemical analysis identified a variety of bioactive compounds in the different solvent extracts of *C. africana* leaves. Notably, saponins and flavonoids, recognized for their potent wound-healing properties, were among the detected constituents [47]. Flavonoids contribute to wound healing by preventing cell necrosis and enhancing vascularization [48]. Moreover, the antimicrobial and antioxidant properties of phenolic compounds and flavonoids play a vital role in protecting against oxidative damage caused by free radicals. These compounds help mitigate microbial infections and reduce inflammation, thereby accelerating the wound-healing process [49]. Tannins further support wound healing by stimulating fibroblast proliferation and migration into the wound site. Additionally, their antibacterial properties help prevent infections, enhancing the overall healing process [50]. Saponins aid in wound recovery by exerting anti-inflammatory effects, facilitating keratinocyte migration for re-epithelialization, and promoting collagen deposition [51]. Similarly, terpenoids significantly enhance wound healing due to their astringent and antimicrobial characteristics [52]. The wound-healing effects of these phytochemicals can be attributed to their individual actions or synergistic interactions [53].

## Conclusion

The study findings support the traditional use of *C. africana* Lam as a wound-healing remedy. Higher concentrations of the 80% methanol, chloroform, and methanol fractions demonstrated significant wound-healing effects, confirming the plant’s efficacy in this regard. The results suggested that less polar solvents are more effective in extracting components with potent wound-healing activity. The wound-healing effects of plant leaf extracts are likely attributed to the presence of secondary metabolites such as flavonoids, saponins, tannins, terpenoids, alkaloids, and phenols.
